# Density Functional Theory, Molecular Docking Study, and *In Vitro* Antioxidant Activity of Cinnamic Acid Isolated From *Piper betle* Leaves

**DOI:** 10.1155/bri/1691257

**Published:** 2025-06-17

**Authors:** Sefren Geiner Tumilaar, Ari Hardianto, Hirofumi Dohi, Dikdik Kurnia

**Affiliations:** ^1^Department of Chemistry, Faculty of Mathematics and Natural Science, Padjadjaran University, Sumedang, West Java, Indonesia; ^2^Graduate School of Horticulture, Chiba University, 1-33 Yayoi Inage-Ku, Chiba, Japan

**Keywords:** antioxidant, cinnamic acid, density functional theory, molecular docking, *Piper betle*

## Abstract

*Piper betle* is an edible plant known for its potent antioxidant activity. Among its phenolic constituents, cinnamic acid has been identified as a key compound contributing to this bioactivity. Although cinnamic acid is a well-known molecule, this study is the first to report its isolation from *P. betle* leaves, contributing valuable insights into the chemotaxonomy and phytochemical profile of the species. The aim of this research is to isolate cinnamic acid from the methanol extract of betel leaves and evaluate its antioxidant activity using DPPH and nonenzymatic mimic superoxide dismutase (mSOD) assays. Furthermore, computational analyses were performed using density functional theory (DFT) to assess the antioxidant properties, and molecular docking studies were conducted to investigate the interaction mechanisms of cinnamic acid and its derivatives with several enzymes. The results obtained that cinnamic acid had a strong antioxidant activity with IC_50_ value using the DPPH and mSOD methods of 76.46 and 36 μg/mL, respectively. The analysis used DFT studies of reactive cinnamic acid as seen from the values of several global descriptive parameters. The deviation in the energy gap from E_HOMO_ and E_LUMO_ is quite small, which is 0.0205 eV. Based on the molecular docking results, cinnamic acid ligands and its derivatives act on the amino acid active sites against xanthine oxidase (XO), NADPH oxidase (NO), cytochrome P450 (CP450), and lipoxygenase (LO) receptors although the binding affinity values are not stronger than the positive control for these four receptors. Therefore, cinnamic acid and its derivatives can be used as a compound to counteract free radicals or as an antioxidant.

## 1. Introduction

*Piper betle* as edible plant is one type of plant that is included in more than 1500 species of the *Piperaceae*. This plant is cultivated in areas with tropical climates and high rainfall such as Indonesia, China, India, and some areas of East Africa [1, 2]. In Indonesia and China, this plant has been used for traditional medicine since ancient times [3]. Several studies have reported that this plant has antioxidant, antimicrobial, antidiabetic, and antifungal activities [4–7].

The secondary metabolites present in *P. betle* include phenols, flavonoids, saponins, alkaloids, phenols, tannins, terpenoids, and steroids [8, 9]. Currently, several bioactive compounds have been isolated and identified from *P. betle* such as 4-*ρ*-coumaroylquinic acid and 3-*ρ*-coumaroylquinic acid [10].

Cinnamic acid belongs to a group of phenolic compounds found in various plants [11, 12]. *Trans*-cinnamic acid and *cis*-cinnamic acid are isomers of cinnamic acid. These two isomers are formed due to the presence of an acrylic acid group on the phenyl ring [13, 14]. Cinnamic acid and its derivatives have several pharmacological activities such as antidiabetic [15], anticancer [16], and hepatoprotective [17]. These biological activities make cinnamic acid an important and promising candidate for therapeutic applications in managing oxidative stress, preventing chronic diseases, and enhancing health. Additionally, cinnamic acid and its derivatives have shown potential in protecting cells from damage caused by free radicals, which are implicated in aging, cardiovascular diseases, and cancer. Its natural occurrence in plants also makes it a valuable target for natural product research, potentially offering safer, plant-based therapeutic options. In addition, cinnamic acid exhibits antimicrobial properties, which could contribute to natural food preservation, reducing reliance on synthetic preservatives and providing safer options for extending food shelf life. Some previous studies also revealed that these compounds have a relatively strong antioxidant activity [18]. This antioxidant activity occurs because these compounds determine radical chain reactions by donating electrons and reacting with radicals to form stable products [19].

The identification of cinnamic acid as an antioxidant in *P. betle* leaves marks a significant milestone in natural product chemistry. Its potent antioxidant properties and diverse applications underscore its importance in medicinal research and various industries. Ongoing research is likely to uncover further benefits and applications of cinnamic acid and its derivatives, solidifying its role as a key natural antioxidant. Isolation of cinnamic acid from *P. betle* leaves was reported for the first time in this study, contributing to the phytochemical characterization of this plant. While cinnamic acid is a well-known isolation molecule in other plants, its isolation from this specific edible plant (*P. betle*) had not been reported previously. Combined in vitro and in silico antioxidant analyses suggest it may act as a potential antioxidant agent by stabilizing free radicals and interacting with oxidative stress-related enzymes.

Natural product-based drug discovery research has been conducted to find lead compounds as new antioxidants. *P. betle* contains important compounds that can be used as a source of antioxidants [20]. Antioxidants can counteract the oxidative process by nitrogen and oxygen species of biomolecules that have caused oxidative stress [21]. Free radical accumulation can damage membranes and cells and cause other oxidative damage [22]. Therefore, chemical compounds derived from natural ingredients can scavenge free radicals to minimize other damage in the body.

Antioxidant activity of compounds can be evaluated using various in vitro methods such as 2,2-diphenyl-1-picrylhydrazyl radical scavenging (DPPH) and mimic superoxide dismutase (mSOD) nonenzymatic [23, 24]. Currently, to aid and speed up, studies conducted in laboratory settings (in vitro studies). By evaluating the molecular structure computationally, scientists can predict how compounds might behave or interact in biological systems, which can streamline and enhance the experimental process [25]. This study focuses on the isolation and identification of cinnamic acid from *P. betle* leaves and the evaluation of its antioxidant activity using DPPH and nonenzymatic mimic superoxide dismutase (mSOD) assays. Subsequently, the antioxidant properties were evaluated using computational methods, including density functional theory (DFT) and molecular docking analyses.

In addition to reporting, for the first time, the isolation of cinnamic acid from *P. betle* leaves, the novelty of this study also lies in the comprehensive investigation of its mechanism of action through in silico approaches, specifically using DFT and molecular docking analyses. These computational techniques allowed us to explore the compound's electron donor/acceptor behavior and its interaction with key antioxidant-related enzymes, which has not been previously reported in the context of cinnamic acid derived from *P. betle*.

## 2. Materials and Methods

### 2.1. Materials

The leaves of *P. betle* were collected from a local farmer in Bandung, West Java, Indonesia, in February 2019. The specimen was identified by botanist and deposited at the Laboratory of Plants Taxonomy, Department of Biology, Universitas Padjadjaran, Indonesia, with number of registration 40/HB/02/2019. In the extraction and purification stages, analytical-grade organic solvents such as methanol, n-hexane, ethyl acetate, and distilled water were used, while chemicals for spectroscopic analysis were used at the proanalytic (p.a) level. Column chromatographic separations were performed on silica gel 60 (63–200 μm and 200–500 μm) (Merck, Darmstadt, Germany) and octadecyl silane (ODS) RP-18 (40–63 μm) (Merck). Thin-layer chromatography (TLC) was performed using Silica G 60 F_254_ (0.25 mm, Merck) plate and ODS RP-18 F_254_S (Merck, Darmstadt, Germany) and visualized by 10% (v/v) H_2_SO_4_ in ethanol.

The DFT and molecular docking calculations were performed using a computing system equipped with a single computer having the following hardware specifications: Intel Core i5 CPU (@2.30 GHz), Random Access Memory (RAM) 4 GB and Graphic Card (Intel HD Graphic 520) as well as all windows-based software in the form of applications Marvin Sketch, Marvin View, PubChem (https://PubChem.ncbi.nlm.nih.gov), GaussView 6.0, Gaussian 9.0, Protein Data Bank (https://www.rcsb.org//pdb), ProTox predictor (https://tox.charite.de/protox3/), BIOVIA Discovery Studio Visualizer 2020, and AutoDock Tools.

### 2.2. Instruments

The structures of the compounds were characterized by MS, UV, FT-IR, ^13^C NMR, ^1^H NMR, HMQC, ^1^H-^1^H COSY, and HMBC spectra. MS spectrometer (Waters Acquity UPLC system coupled with a triple quadrupole (UPLC–TQ), Agilent, CA, USA) was used to measure mass spectrum with the electron impact mode. UV spectrometer (8452A Diode Array, Hewlett Packard Palo Alto, USA) was used to measure UV spectrum. The FT-IR spectrum was recorded with a FT-IR Shimadzu 8400 spectrometer (SpectraLab Scientific Inc., Markham, Canada) using KBr disc with CH_2_Cl_2_ as a solvent. ^1^H NMR (500 MHz), ^13^C NMR (125 MHz), and other 2D spectra were recorded with a JEOL ECA 500 spectrometer at 25°C.

Antioxidant activity assay was performed using 96-well microplates (NEST Biotechnology, Wuxi, China) and microplate readers (Biochrom Ltd., Cambridge, UK).

### 2.3. Extraction, Fractionation, Isolation, Characterization

**Figure 1:**
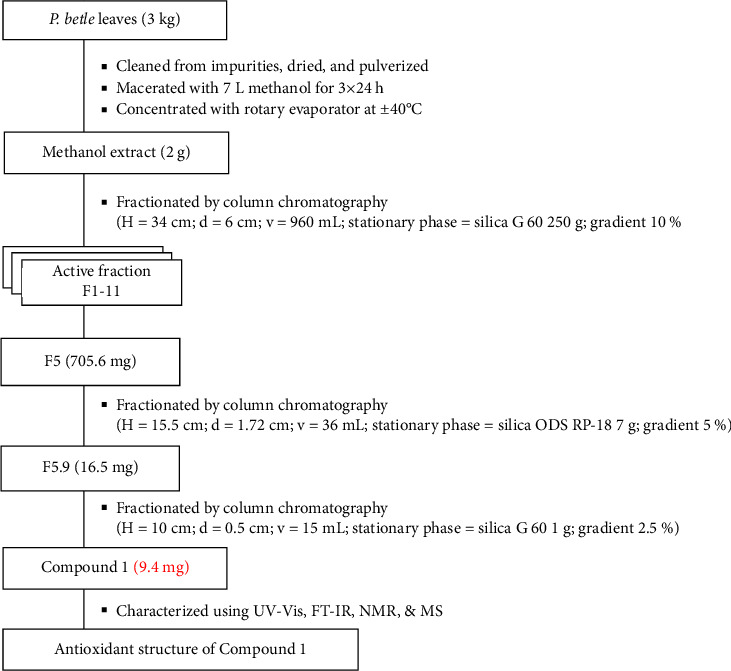
Flowchart extraction and isolation of Compound **1** from *P. betle*.

The collected *P. betle* leaf samples (3 kg) were cut into small pieces, dried, and powdered using a mechanical grinder and then obtained 0.37 kg of concentrated methanol extract using the maceration method for 3 × 24 h. Resulting macerate was filtered using a filter funnel, and the filtrate was collected. The filtrate was then concentrated under diminished pressure using a rotary evaporator at 40°C. Crude extract (2 g) was fractionated into eleven fractions (1–11) with silica gel 60 and a solvent mobile mixture n-hexane and ethyl acetate with a 10% gradient. Each fraction that will be formed is tested for antioxidant activity and looked for fractions that are acting as antioxidants. Fraction 5 (705.6 mg) was subjected to column chromatography on silica ODS gel (7 g) eluted with 5% methanol and water to yield eleven fractions. Fraction 5.9 (16.5 mg) was rechromatographed over the silica gel 60 (1 g) eluted with 2.5% n-hexane and ethyl acetate. Compound **1** (9.4 mg) was purified as cinnamic acid (**1**) from this fraction. Then, the characterization of Compound **1** was analyzed using spectrophotometer UV-VIS, FT-IR, NMR, and MS. The flowchart extraction and isolation of Compound **1** from *P. betle* are shown in Figure 1.

### 2.4. DPPH Free Radical Scavenging Assay

The sample (5 mg) was dissolved and diluted up to 5 mL of methanol. From this solution, various concentrations of 0–80 μg/mL were prepared. 0.6 mL of 0.001 M of DPPH was added to it [26]. Methanol and sample were put into test tubes according to the composition listed in Table 1. All these solutions were kept in a dark room for 30 min. The mixture was examined for antioxidant activity using a microplate reader at a wavelength of 517 nm and calculated the percent inhibition using the following equation [27]:

(1) $$\begin{array}{c} \%\text{ Inhibition}=\frac{A_{\text{control}}-A_{\text{sample}}}{A_{\text{control}}}\times 100. \end{array}$$

### 2.5. mSOD Nonenzymatic Assay

Antioxidant activity testing using mSOD nonenzymatic assay according to the method is described by Deawati et al. [24]. This method consists of several steps including preparation of sample stock solution, preparation of working solutions 1 and 2, preparation of test solution in a microplate (96-well), irradiation of microplate containing test solution, measurement of absorbance of the test solution, and calculation of % inhibition and IC_50_ value. The sample solution was prepared by dissolving it in methanol at a predetermined concentration. The next step is the preparation of working solutions 1 and 2. Working solution 1 consists of aquabidest and phosphate buffer pH 7.41 M, NBT 10 mM, TEMED 0.1 M, and riboflavin 0.5 mM, while working solution 2 consists of the same solution, but without the addition of riboflavin solution. The sample solution, working solutions 1 and 2, and methanol that had been prepared were then pretreated on a microplate with different concentration variations. Concentration variations were carried out by microdilution and homogenized using micropipettes. Microplate containing the test solution was irradiated with a lighting box for approximately 10 min. Next, the absorbance was measured at 550 nm using a microplate reader.

All antioxidant assays included appropriate solvent controls, in which methanol—the solvent used for sample preparation—was tested alone at equivalent volumes. These controls confirmed that methanol did not exhibit any significant antioxidant activity (inhibition < 5%) and was therefore considered innocuous within the experimental conditions.

### 2.6. Molecular Structure Activity Using DFT

This computerized test uses software with the Becke, 3-parameter, Lee–Yang–Parr (B3LYP)/6-31G (2d, 2p) basis set in the gas phase [28, 29]. The structure of the test compound cinnamic acid (**1**) and quercetin as a positive control was downloaded on the web page https://pubchem.ncbi.nlm.nih.gov/ by selecting the structure at physiological pH (pH 7.4) [25]. Structure optimized in the neutral state using Gaussian 9.0 and GaussView 6.0 software. The optimization results in (.log) format are recalculated to obtain the frequency and single point energy (SPE) values. This step is also repeated on the structure of the test compound in the anion and cation state until the SPE value of each charge (neutral, anion, and cation) is obtained [30]. The three SPE values are used to determine the global descriptive parameter (GDP) value. The GDP value is then used to calculate electron-donating power (*ω*^−^), electron-accepting power (*ω*^+^), index donor (Rd), and index acceptor (Ra). Test compounds that have been calculated using the DFT method are then visualized using GaussView 6.0 software to determine the energy value and distribution of the highest occupied molecular orbital (HOMO) and lowest unoccupied molecular orbital (LUMO) until the energy gap value is obtained [31, 32]. The equations for determining SPE (ionization potential [I] and electron affinity [A], GDP, electron-donating power [*ω*^−^], and electron-accepting power [*ω*^+^]) are given as follows [25]:

(2) $$\begin{array}{c} I=E_{\text{cation }}-E_{\text{netral}}, \end{array}$$

(3) $$\begin{array}{c} A=E_{\text{netral }}-E_{\text{anion}}, \end{array}$$

(4) $$\begin{array}{c} \eta =\left(I-A\right)\cdot \frac{1}{2}, \end{array}$$

(5) $$\begin{array}{c} \chi =\left(I+A\right)\cdot \frac{1}{2}, \end{array}$$

(6) $$\begin{array}{c} S=\frac{1}{\left(2\left(I-A\right)\cdot \left(1/2\right)\right)}, \end{array}$$

(7) $$\begin{array}{c} \mu =-\left(\left(I+A\right)\cdot \frac{1}{2}\right), \end{array}$$

(8) $$\begin{array}{c} \omega =\frac{{\left.\left(-\left(I+A\right)\cdot \left(1/2\right)\right)|\text{⁣}\right)}^{2}}{2}, \end{array}$$

(9) $$\begin{array}{c} \omega -=\frac{{\left(3I+A\right)}^{2}}{16\left(I-A\right)}, \end{array}$$

(10) $$\begin{array}{c} \omega +=\frac{{\left(I+3A\right)}^{2}}{16\left(I-A\right)}, \end{array}$$

(11) $$\begin{array}{c} \text{Ra}=\frac{\omega_{l}^{+}}{\omega_{f}^{+}}, \end{array}$$

(12) $$\begin{array}{c} \text{Rd}=\frac{\omega_{l}^{-}}{\omega_{f}^{-}}. \end{array}$$

### 2.7. Molecular Docking Study

**Figure 2:**
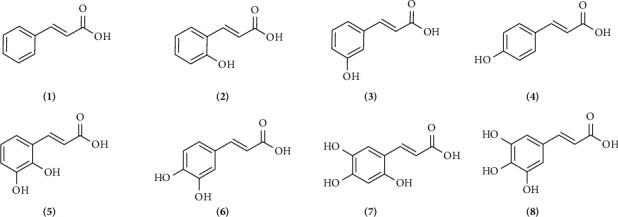
Chemical structures of cinnamic acid (**1**) and its derivatives that belong to the cinnamic group such as *o*-coumaric cinnamate (**2**), *m*-coumaric cinnamate (**3**), *p*-coumaric acid (**4**), 2,3-dihydroxycinnamic acid (**5**), caffeic acid (**6**), 2,4,5-trihydroxycinnamic acid (**7**), and 3,4,5-trihydroxycinnamic acid (**8**).

**Figure 3:**
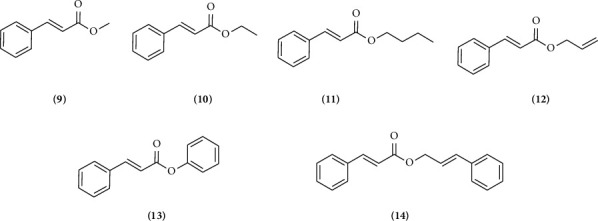
Chemical structures that belong to the ester cinnamic group such as methyl cinnamate (**9**), ethyl cinnamate (**10**), butyl cinnamate (**11**), allyl cinnamate (**12**), phenyl cinnamate (**13**), and cinnamyl cinnamate (**14**).

The selection of compounds used as ligands in the docking process for this study was based on compounds successfully isolated from *P. betle* leaves, namely, cinnamic acid (**1**) and compared with derivative compounds which are divided into cinnamic group (Figure 2) and ester cinnamic group (Figure 3). These compounds were obtained from the PubChem web server (https://pubchem.ncbi.nlm.nih.gov) as part of a digital library search. Each ligand's name was entered into the search field to do the search. The files for each ligand were then downloaded and stored. Using AutoDock Tools, the ligands were opened in .pdb format. Torque was then adjusted by identifying the root and making necessary adjustments. After that, the modified file was saved in .pdbqt format. Lipinski's rule of five was used to compute the characteristics of the active compounds using the ProTox predictor (https://tox.charite.de/protox3/) [33].

Four receptors were selected as targets for antioxidant discovery: xanthine oxidase (XO) (PDB ID 3NVY) [34], NADPH oxidase (NO) (PDB ID 2CDU), cytochrome P450 (CP450) (PDB ID 1OG5), and lipoxygenase (LO) (PDB ID 1N8Q) [35]. Receptor three-dimensional (3D) structures were downloaded in .pdb format from the Protein Data Bank (https://www.rcsb.org//pdb) [36]. In the docking process, these proteins served as receptors. After using BIOVIA Discovery Studio Visualizer 2020 to access the data, water molecules and any remaining ligands bound to the receptors were eliminated. Next, the receptors were stored in a.pdb file format. Using AutoDock Tools [37], hydrogen atoms were added to the receptors, and the files were then stored in the .pdbqt format.

Using AutoDock Tools, the positions of the amino acids that function as active sites on the receptor where the ligands dock were found. During this process, a particular docking method was used to produce a 3D map of the grid box. The 3D coordinates for XO receptor docking were *x*: 39.367, *y*: 21.888, and *z*: 20.012; NO receptor 3D coordinates were *x*: 2.500, *y*: −0.555, and *z*: −0.797; and CP450 receptor 3D coordinates were *x*: −20.236, *y*: 86.761, and *z*: 38.657, while LO receptor 3D coordinates were *x*: 21.864, *y*: 2.184, and *z*: 18.909. Each docking was configured using 40 user-specified grid points.

The docking process was carried out using Autodock4. The Autodock4 program was executed via the command prompt. The docking calculation results were shown in an output file in Notepad. By choosing the pose with the highest affinity, represented by the most negative Gibbs free energy of binding, the ligands' docking conformation was ascertained. The visualization of the involved amino acid residues and their positions was conducted using the BIOVIA Discovery Studio Visualizer 2020 application, allowing for analysis in various conformations.

## 3. Result and Discussion

### 3.1. Identification of Compound 1

Compound **1** (9.4 mg) has been isolated from *P. betle*, which was obtained as a pale brown-yellowish oil. The ion peak at m/z 147.14 [M-H]^−^ in negative ion MS high-resolution mass spectrometry shows that this compound's molecular weight is 148. The origin MS is provided as Supporting Figure S8 in the Supporting Information. Compound **1** can fluoresce in UV light 254, does not fluoresce in UV light 356, and was not visualized by 10% H_2_SO_4_ stain spotter. Based on the characterization of the UV-Vis spectrophotometer, Compound **1** with a concentration of 20 ppm showed a peak at a wavelength of 215 nm indicating the presence of an C=C band with π − π^∗^ electron transitions [38]. Furthermore, it shows a peak at a wavelength of 271 nm indicating the presence of aromatic rings with π − π^∗^ electron transitions [39]. The UV-Vis spectrum data are shown in Supporting Figure S1, with its interpretation detailed in Supporting Table S1 of the Supporting Information. The FT-IR spectrum of Compound **1** showed the presence of C-H sp^3^ (2925.37 cm^−1^), C=O (1691.62 cm^−1^), C=C (1495.03 cm^−1^), and C-O (1286.78 cm^−1^). These data have a shift that is similar to the peak of the cinnamic acid (**1**) FT-IR spectrum that has been published in previous studies [40]. The FT-IR spectrum data are presented in Supporting Figure S2 of the Supporting Information.

**Figure 4:**
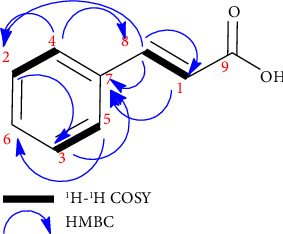
^1^H-^1^H COSY and HMBC correlations in Compound **1**.

Based on analysis of ^13^C NMR and DEPT 135° spectra, Compound **1** has nine carbon atoms including seven methyne sp^2^ carbons (*δ*_*c*_ 118.4, C-1; 127.8, C-2; 127.8, C-3; 128.6, C-4; 128.6, C-5; 129.9, C-6; and 144.6 ppm, C-8), one sp^2^ quaternary carbon (*δ*_*c*_ 134.5 ppm, C-7), and one carboxylic carbon (*δ*_*c*_ 169.4 ppm, C-9), as demonstrated in Supporting Figure S3, with its interpretation detailed in Supporting Table S2 of the Supporting Information. The HMQC spectrum is used to confirm the ownership of the ^13^C-^1^H [41]. The complete data can be seen in the original HMQC spectrum provided in Supporting Figure S4 of the Supporting Information. The ^1^H-NMR spectrum of Compound **1** (in CD_3_OD) shows protons for methyne sp^2^ at *δ*_*H*_ 6.46 (1H, d, *J = *16 Hz, H-1), 7.38 (2H, m, H-2 & H-3), 7.56 (2H, m, H-4 & H-5), and 7.63 (1H, d, *J* = 16 Hz, H-8). Based on the spectrum, the H-2, H-3, H-4, and H-5 protons show a typical shift for the benzene ring and judging from the J-coupling value, and the H-1 and H-8 protons show a typical shift of the proton at double-bonded carbon, as shown in Supporting Figure S5, with its interpretation detailed in Supporting Table S3 of the Supporting Information. Furthermore, to strengthen the reliability of the suspected structure, spectrum analysis of the HMBC and ^1^H-^1^H COSY was carried out. The ^1^H-^1^H COSY spectrum is to determine the relationship between ^1^H and ^1^H with the distance of three bonds. The complete data can be seen in the original ^1^H-^1^H COSY spectrum provided in Supporting Figure S6 of the Supporting Information, while the HMBC spectrum is used to determine the correlation of protons and carbons with a distance of three to four bonds. The complete data can be seen in the original HMBC spectrum provided in Supporting Figure S7 of the Supporting Information. The ^1^H-^1^H COSY and HMBC correlations are shown in Figure 4.

Finally, compared to the spectrum data of previously published references, we found that the chemical shift of the carbon and proton of Compound **1** are not significantly different from that of cinnamic acid (**1**) [42, 43]. Notably, this is the first time that this compound has been isolated from *P. betle* leaves, adding a new dimension to the phytochemical profile of this plant.

### 3.2. In Vitro Antioxidant Activity

Compound **1** as cinnamic acid isolated from *P. betle* leaves was tested for its antioxidant activity using the DPPH and mSOD methods as shown in Table 2. Quercetin was used as a positive control and compared with *P. betle* methanol extract. In the DPPH test, the radical source comes from DPPH which will be stabilized through electron donors by antioxidant compounds. The more DPPH is stabilized, and the maximum wavelength absorption of DPPH will decrease, which can be measured at a wavelength of 517 nm [44]. The principle of the mSOD nonenzymatic method is to study the ability of a sample to inhibit the active redox indicator reduction process by O_2_•^−^ species until the IC_50_ value is obtained which is measured at *λ*_max_ 550 nm [24].

According to Triana et al. [45], antioxidant activity can be classified based on the IC_50_ value. This parameter explains that the smaller the IC_50_ value, the stronger the antioxidant activity. IC_50_ less than 50 μg/mL is classified as very strong, 50–100 μg/mL is classified as strong, 101–150 μg/mL is classified as moderate, and more than 150 μg/mL is classified as weak antioxidant activity. The IC_50_ values for cinnamic acid (**1**) were 76.46 ± 2.85 μg/mL (DPPH) and 34 ± 1.73 μg/mL (mSOD), based on three independent experiments. IC_50_ value of cinnamic acid (**1)** has antioxidant activity which is classified as strong by the DPPH and very strong by the mSOD nonenzymatic method. This happens because cinnamic acid (**1**) is affected by the presence of a benzene aromatic ring, the hydroxyl group (OH) attached to the benzene aromatic ring (phenolic hydroxyl) at C-9 which plays an important role in influencing antioxidant activity [46, 47]. The presence of the hydroxyl group of carboxylic acids plays an important role in the structure–activity relationship (SAR) of antioxidants because they have a good ability to donate electrons [48]. The benzene ring has a conjugated carbon double bond that is able to delocalize electrons so that it can stabilize free radicals from DPPH into its nonradical form (DPPH-H) and can stabilize superoxide anion radicals (O_2_•^−^) [49].

However, as shown in Table 2, it should be stated that the positive control compound (quercetin) and methanol extract were more active than cinnamic acid (**1)**. From these results, quercetin, used as a positive control, exhibited significantly stronger antioxidant activity with IC_50_ values of 3.88 ± 0.21 μg/mL (DPPH) and 5 ± 0.19 μg/mL (mSOD). The methanol extract of *P. betle* also showed potent activity, with IC_50_ values of 6.87 ± 0.31 μg/mL (DPPH) and 9 ± 0.26 μg/mL (mSOD). It is evident that quercetin has the highest antioxidant activity (lowest IC_50_ value) among the tested samples, followed by the methanol extract and then Compound **1**. Quercetin's superior activity is consistent with its known strong free radical scavenging ability due to its multiple hydroxyl groups, which can donate hydrogen atoms effectively. The methanol extract, likely containing a mixture of various bioactive compounds, also shows significant activity, albeit weaker than quercetin but much stronger than cinnamic acid (**1**). Similar to the DPPH assay, quercetin again shows the highest activity in the mSOD assay, with an IC_50_ value of 5 ± 0.19 μg/mL. This suggests that quercetin not only has potent radical scavenging activity but also effectively neutralizes superoxide radicals. The methanol extract demonstrates moderate activity (IC_50_: 9 ± 0.26 μg/mL), indicating the presence of compounds that can mimic or support SOD activity. Cinnamic acid (**1)**, while more active in the mSOD assay than in the DPPH assay, still exhibits relatively lower antioxidant activity (IC_50_: 34 ± 1.73 μg/mL) compared to quercetin and the methanol extract. The findings suggest that while cinnamic acid (**1)** has some antioxidant potential, it is not as effective as quercetin or the methanol extract. This could limit its application as a stand-alone antioxidant agent. However, it may still contribute beneficially when used in combination with other antioxidants due to potential synergistic effects. The methanol extract's relatively strong performance warrants further investigation. Identifying and isolating the individual components responsible for its activity could lead to the development of novel antioxidant agents. All assays included methanol as a solvent control, which showed negligible antioxidant activity (inhibition < 5%), confirming that the solvent did not interfere with the results.

Although the methanol extract showed stronger overall activity than the isolated compound, the focus on cinnamic acid (**1**) was due to its consistent presence in bioactive fractions and its known pharmacophore features. This compound serves as a marker and provides mechanistic insight for future standardization or bio-guided fractionation studies. Cinnamic acid (**1**) is a secondary metabolite compound which is one of the largest phenolic acid groups [50]. There are three proposed mechanisms by which phenolic compounds exert free radical scavenging activity. The first one uses abstracted hydrogen atoms as antioxidants. The second step is the formation of ArOH•^+^ radical cations by transferring electrons from antioxidants to deprotonated free radicals to produce ArO• radical and ROH. In the third stage, the phenolic compound is deprotonated and an electron transfer occurs to RO• from the phenoxide anion [22].

### 3.3. DFT Study

DFT calculation is used to determine the prediction of antioxidant and antiradical reactivity of compounds involving electron donors and acceptors [51]. In this method, cinnamic acid (**1**) and quercetin as positive controls are optimized for geometry using the B3LYP/6-31G (2d,2p) basis set in the gas phase and then proceed with calculating the vibrational frequency using the same basis set to obtain a SPE value for each compound [52]. The optimization process functions to change the bond length and angle of each structure so that it approaches the actual optimal bond length and angle [32]. However, previously, the cinnamic acid (**1**) structure was selected at a physiological pH of 7.4. It aims to see changes in the geometry of the structure in the protonated state [53].

GDP consists of hardness (*η*), electronegativity (*χ*), softness (*S*), chemical potential (*μ*), and electrophilicity (*ω*). The larger the *η* value indicates that the compound has good configurational stability [54]. The values of *η* and *S* also determine the reactivity of a molecule and its ability to donate electrons. The smaller *S* value indicates that the molecule has good reactivity. The more negative the value of *μ* indicates that the molecule has high stability. The smaller the *ω* value indicates that the molecule has high reactivity because it is easier to donate electrons [55].  Table 3 shows that cinnamic acid (**1**) has higher stability than quercetin because it has the largest *η* value of 9.56 eV and a smaller *μ* value of 0.05 eV. The reactivity value of cinnamic acid (**1**), which is 5.03 eV, is not better than the reactivity value of quercetin, which has a value of 0.13 eV, because cinnamic acid (**1**) has quite a large S value compared to quercetin.

**Figure 5:**
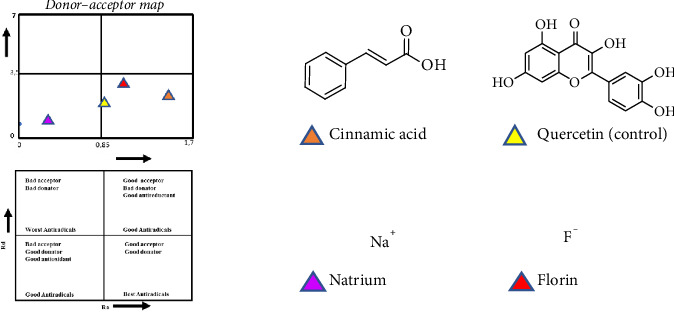
Diagram for determining antioxidant activity using DAM [56].

Analysis using the donor–acceptor map (DAM) is a continuation of the results of calculations using the GDP method. The parameters in DAM are compared with the calculation results from natrium (Na) and florin (F) atoms. The Na atom represents an atom that has the ability to be a good electron donor. Meanwhile, the F atom represents an atom that has the ability to be a good electron acceptor [56]. Based on the results of the analysis using DAM (Figure 5), it shows that cinnamic acid (**1**) has good antiradical abilities. A compound or molecule can be said to be a good antiradical when the molecule can be a good donor and acceptor [57].

**Figure 6:**
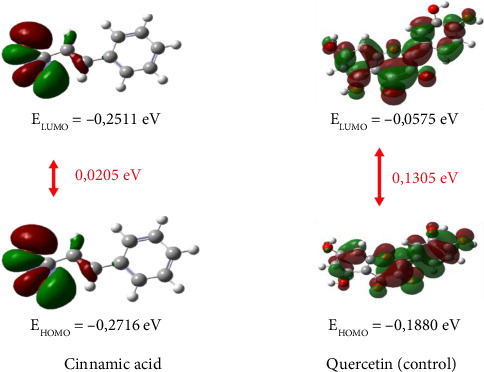
HOMO-LUMO energy gap visualization results from cinnamic acid (**1**).

Frontier molecule orbital (FMO) is one of the parameters in determining a molecule as an electron acceptor–donor and predicting its reactive position in the *π* electron system [58]. FMO is formed due to the HOMO and LUMO [59]. This analysis is related to the energy gap obtained from the HOMO–LUMO energy. The energy gap determines the reactivity of a molecule. The smaller the energy gap value, the more reactive the molecule is in donating and accepting electrons. This FMO analysis can also show the distribution of orbitals in a molecule, which indicates the location of attack by radicals on the molecule [60]. The results of the HOMO-LUMO analysis of cinnamic acid (**1**) can be seen in Figure 6. Based on the results, cinnamic acid (**1**) has a smaller energy gap than quercetin. This shows that cinnamic acid (**1**) is more reactive than quercetin as well as being a good donor and acceptor.

These computational methods allowed us to explore a detailed investigation of the electronic properties of cinnamic acid (**1**), including its behavior as an electron donor and acceptor. Through DFT calculations, we obtained key GDPs—such as ionization potential, electron affinity, electronegativity, hardness, softness, and electrophilicity index—that collectively describe the compound's chemical reactivity and stability. Furthermore, analysis of the HOMO and LUMO provided insight into its frontier molecular orbital distribution and energy gap, which are critical for understanding its potential antioxidant and radical scavenging capabilities.

### 3.4. Molecular Docking Study

Molecular docking is a computational technique employed to predict the interactions between two molecules, such as a ligand and a receptor, at the atomic scale [61, 62]. This method aids in understanding how a ligand binds to a receptor's active site and evaluates the ligand's affinity and optimal orientation within that site. Molecular docking is frequently utilized in drug design to identify and refine potential therapeutic compounds [63].

In this study, there are four receptors like XO, NO, CP450, and LO were selected as targets for receptor docking. XO has a close relationship with free radical production in the body. During its catalytic activity, XO receptor converts hypoxanthine to xanthine and then xanthine to uric acid. This process not only produces uric acid but also generates reactive oxygen species (ROS), including superoxide radical (O_2_•) and hydrogen peroxide (H_2_O_2_) [64]. Therefore, this enzyme needs to be inhibited in the body.

**Figure 7:**
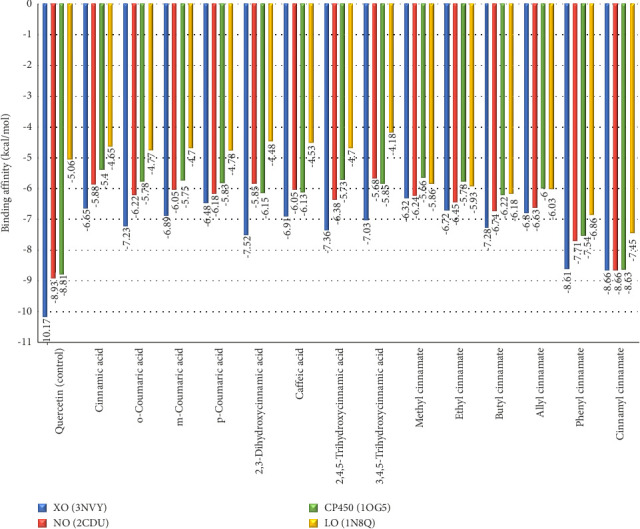
Histogram showing the binding affinity of xanthine oxidase (XO), NADPH oxidase (NO), cytochrome P450 (CP450), and lipoxygenase (LO) receptors with cinnamic acid (**1**) and its derivatives although all cinnamic acid ligands and their derivatives do not exhibit higher binding affinity compared to the control ligand (quercetin), and the amino acid residues involved in the binding indicate that all these ligands bind to the active site. These ligands inhibit the performance of this receptor with various types of binding. Thus, cinnamic acid (**1**) ligands and their derivatives still have the potential as inhibitors of the XO receptor activity.

Figure 7 shows the binding affinity results of cinnamic acid (**1**) and its derivatives against the XO, NO, CP450, and LO receptors. Binding affinity is a measure of the strength of the interaction between two molecules, such as a ligand and a receptor, in a molecular complex. Binding affinity indicates how strongly a ligand binds to a receptor. The more negative the binding affinity value, the higher the binding affinity between the ligand and the receptor, indicating a stronger interaction [65]. Based on Figure 7, the difference in binding affinity between cinnamic acid (**1**) and its derivatives bound to one hydroxyl group (*o*-coumaric acid (**2**), *m*-coumaric acid (**3**), *p*-coumaric acid (**4**)) can be explained through several chemical factors and molecular interactions that affect the binding affinity with the target protein. *o*-Coumaric acid (**2**) (−7.23 kcal/mol): The *ortho* position of the -OH group allows for optimal hydrogen bond formation with residues around the active site, providing the highest binding affinity among these isomers. *m*-Coumaric acid (**3**) (−6.89 kcal/mol): The -OH group at the *meta* position may also interact via hydrogen bonding, although it may be slightly less optimal than with the *ortho* position. *p*-Coumaric acid (**4**) (−6.48 kcal/mol): The -OH group in the *para* position is often less efficient at forming hydrogen bonds with residues in the active site compared to the *ortho* or *meta* position. Cinnamic acid (**1**) has a higher binding affinity than *p*-coumaric acid (**4**) (−6.55 kcal/mol) but lower than *o*-coumaric acid (**2**) and *m*-coumaric acid (**3**) because it lacks hydroxyl groups that can interact directly through hydrogen bonding. When compared to 2,3-dihydrocinnamic acid (**4**), it has a binding affinity of −7.52 and caffeic acid (**5**) −6.91 kcal/mol. This is because 2,3-dihydrocinnamic acid has better molecular flexibility and van der Waals interactions and a more optimal fit with the protein active site [66], while caffeic acid has a lower binding affinity despite having two hydroxyl groups due to the possibility of steric hindrance and lack of molecular flexibility compared to 2,3-dihydrocinnamic acid, which can reduce the effectiveness of interactions with the active site. Nevertheless, these two molecules with the addition of two -OH groups still bind better than cinnamic acid (**1**). While the binding affinity value of two ligands substituted with three -OH groups, 2,4,5-trihydroxycinnamic acid (7) is −7.36 kcal/mol and 3,4,5-trihydroxycinnamic acid (8) is −7.03 kcal/mol. The value is stronger than cinnamic acid (**1**). The small difference in binding affinity between 2,4,5-trihydroxycinnamate and 3,4,5-trihydroxycinnamate is most likely due to a combination of -OH group orientation, hydrogen bond strength and orientation, steric effects, and electron distribution and resonance.

Overall, an increase in the binding affinity of cinnamate derivatives as the size and hydrophobicity of the substituent groups increase, as well as the ability for stronger π − π stacking interactions, is to be expected. Stronger hydrophobic interactions and the ability to form noncovalent interactions such as π − π stacking with aromatic residues in the binding site often increase the stability of the ligand–receptor complex, which is reflected in lower (more negative) binding affinity values. The binding affinity value of the ester cinnamic group increases with the addition of substituents. The following binding affinity values of ester cinnamic group are cinnamyl cinnamate (**14**) −8.66 > phenyl cinnamate (**13**) −8.61 > butyl cinnamate (**11**) −7.28 > allyl cinnamate (**12**) −6.8 > ethyl cinnamate (**10**) −6.72 > methyl cinnamate (**9**) −6.32 kcal/mol.

NO receptor is a primary source of ROS in cells. They are involved in the immune response, particularly in phagocytes, where they produce ROS to kill pathogens [67]. However, excessive ROS production is linked to oxidative stress, which can damage cells and tissues. Inhibiting NO can help reduce oxidative stress, which is implicated in a variety of conditions such as cardiovascular diseases. Certain CP450 isoforms, particularly those involved in drug metabolism (like CP2E1), can produce significant amounts of ROS. Inhibiting these isoforms can reduce ROS generation and mitigate oxidative stress [68], while inhibiting LO receptor reduces the formation of lipid peroxides, which are harmful by-products that can propagate oxidative damage in cell membranes and lipoproteins [69]. This action is similar to how lipid-soluble antioxidants like vitamin E prevent lipid peroxidation.

Based on Figure 7, binding with NO receptor, quercetin as positive control exhibits the highest binding affinity of −8.93 kcal/mol, indicating strong inhibitory potential. Cinnamyl cinnamate (**14**) shows the next highest binding affinity of −8.66 kcal/mol, followed by phenyl cinnamate (**13**) with −7.71 kcal/mol. These compounds may inhibit NO effectively, thereby reducing ROS production and oxidative stress. When binding with CP450 receptor, among the derivatives, cinnamyl cinnamate (**14**) and phenyl cinnamate (**13**) have high binding affinities of −8.63 and −7.54 kcal/mol, respectively. These values suggest they might interact significantly with CP450, potentially modulating oxidative metabolism and preventing the formation of reactive metabolites, while binding with LO receptor cinnamyl cinnamate (**14**) (−7.45 kcal/mol) and phenyl cinnamate (**13**) (−6.86 kcal/mol) again show the highest affinities, suggesting they could effectively inhibit LO, reducing lipid peroxidation and inflammation.

The binding affinities correlate with structural modifications in the cinnamic acid (**1**) derivatives as follows: derivatives with larger, bulkier substituents, such as cinnamyl cinnamate (**14**) and phenyl cinnamate (**13**), show higher binding affinities across all targets. This could be due to increased hydrophobic interactions and steric compatibility within the enzyme active sites. Compounds like caffeic acid (**6**) and tri-hydroxycinnamic acids (**7** and **8**) have moderate binding affinities, likely due to the hydrophilic nature of the hydroxyl groups, which may not favor strong interactions in hydrophobic regions of the enzymes.

**Figure 8:**
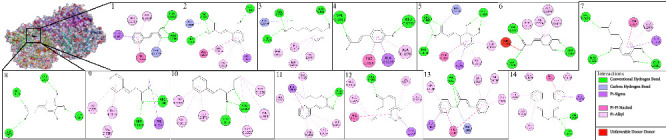
Two-dimensional and three-dimensional configurations of ligand binding for cinnamic acid (**1**) and its derivatives to the xanthine oxidase (XO) receptor (PDB ID: 3NVY).

The active site location of the amino acid residues on the XO receptor (PDB ID: 3NVY) is located at Glu^802^, Arg^880^, Arg^912^, Phe^914^, and Glu^1261^ [70]. Based on Figure 8, cinnamic acid (**1**) and its derivatives bind to five amino acid residues in the active site of the XO receptor through various types of interactions. All these ligands form more than two hydrogen bonds with the amino acid residues of the receptor. The more hydrogen bonds that are formed, the more stable and stronger the binding between the ligand and the receptor, which also helps stabilize the ligand within the receptor active site [71]. Especially, in the binding of cinnamic acid (**1**) ligand and receptor, there are two conventional hydrogen bonds (Thr^1010^ and Arg^880^) and one carbon–hydrogen bond (Phe^1009^). In addition, there are other types of electrostatic bonds that strengthen and stabilize the ligand–receptor bond (Leu^873^, Phe^914^, Val^1011^, and Leu^1014^).

**Figure 9:**
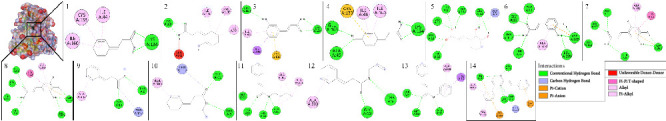
Two-dimensional and three-dimensional configurations of ligand binding for cinnamic acid (**1**) and its derivatives to the NADPH oxidase (NO) receptor (PDB ID: 2CDU).

The active site location of these amino acids is based on the amino acid residues involved in the binding of quercetin as a positive control with NO receptor. Based on Figure 9, all cinnamic acid ligands and their derivatives bind to the active side of the NO receptor which makes the higher inhibition of this receptor. Besides hydrogen bonds, there are also alkyl and pi-alkyl bond types to consider. Alkyl and pi-alkyl bonds are types of noncovalent interactions that are important in determining the affinity and stability of ligand–receptor complexes [72]. In addition, other electrostatic bonds (pi-anions and pi-pi t-shaped) contribute significantly to the stability and affinity of the ligand–receptor complex [73]. Especially, in the binding of cinnamic acid (**1**) and this receptor, there is one hydrogen bond (Lys^134^), and three alkyl and pi-alkyl bonds (Ile^44^, Cys^133^, and Ile^160^).

**Figure 10:**
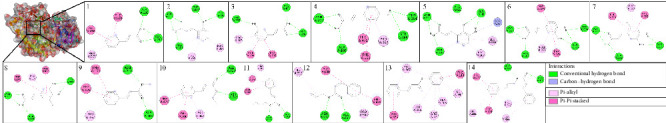
Two-dimensional and three-dimensional configurations of ligand binding for cinnamic acid (**1**) and its derivatives to the cytochrome P450 (CP450) receptor (PDB ID: 1OG5).

**Figure 11:**
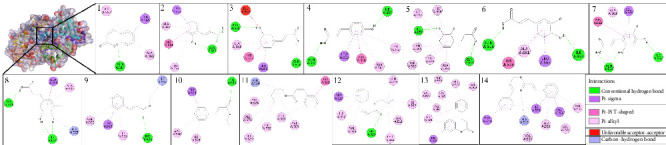
Two-dimensional and three-dimensional configurations of ligand binding for cinnamic acid (**1**) and its derivatives to the lipoxygenase (LO) receptor (PDB ID: 1N8Q).

Binding with the CP 450 receptor shows the active site on the amino acids Arg^97^, Phe^100^, Ala^103^, Gln^214^, Asn^217^, Leu^366^, Pro^367^, Asn^474^, Gly^475^, and Phe^476^. This is demonstrated by the binding of quercetin as a positive control against this receptor. Meanwhile, in the binding with the LO receptor against the positive control, the active site shows amino acids such as Gln^514^, His^518^, His^523^, Leu^565^, Val^571^, Ile^572^, Asp^766^, Val^769^, Leu^773^, and Ile^857^. As shown in Figures 10 and 11, all cinnamic acid and its derivatives act on this active site, indicating that they can inhibit these receptors despite the various types of interactions involved. This suggests that cinnamic acid (**1**) and its derivatives can effectively inhibit these receptors.

The antioxidant activity of cinnamic acid (**1**) is believed to involve several complementary mechanisms: (i) hydrogen atom transfer (HAT) from its phenolic hydroxyl group, (ii) resonance stabilization via its conjugated double bond system, and (iii) inhibition of oxidative enzymes such as XO, NO, CP450, and LO receptors. Additionally, cinnamic acid (**1**) enhances cellular antioxidant responses and suppresses lipid peroxidation.

A strong positive correlation (Pearson's *R* = 0.954) was observed between the calculated GDP and the antioxidant activity as well as the molecular docking scores. This indicates that the electronic properties of cinnamic acid (**1**), as predicted by DFT, are strongly associated with its antioxidant activity, supporting the relevance of computational descriptors in predicting bioactivity.

Inhibition constant (*Ki*) is an important measure in molecular docking and biochemistry that describes how strongly an inhibitor binds to an enzyme or receptor [74]. In the context of molecular docking, it provides an estimate of the binding affinity between the ligand (inhibitor) and the target protein [75]. The smaller the *Ki* value, the stronger the binding of the ligand to the receptor [76]. Based on the result of this study, we try to analyze SAR. In cinnamic acid (**1**) derivatives, the presence and position of hydroxyl groups on the aromatic ring significantly impact enzyme inhibition. Compounds with multiple hydroxyl groups (e.g., 2,3-dihydroxycinnamic acid (**5**), caffeic acid (**6**)) generally show improved inhibition against CP450 and XO, likely due to enhanced hydrogen bonding. Esterification of the carboxyl group (Compounds **9–14**) greatly improves the inhibitory potential against all tested enzymes. The increased hydrophobicity and potentially altered conformation contribute to better interactions with the enzymes. Shorter alkyl chains (e.g., methyl and ethyl cinnamate) generally show weaker inhibition compared to longer or more complex substituents (e.g., phenyl and cinnamyl cinnamate), suggesting that a balance of hydrophobic and hydrophilic interactions is crucial for optimal binding. The substituent of phenyl and cinnamyl in Compounds **13** and **14**, respectively, results in very low *Ki* values across all enzymes tested, highlighting their strong inhibitory potential, possibly due to enhanced *π* − *π* stacking and hydrophobic interactions with the enzyme's active site.

Lipinski's rule of five is a guideline used to predict the pharmacokinetic properties (such as absorption, distribution, metabolism, and excretion) of pharmaceutical compounds, especially those related to oral bioavailability [77, 78]. This helps ensure that the chosen compound is not only effective in binding to the target but also has the potential to be a good drug [79, 80]. There are five parameters that must be adhered to in this rule, namely, (1) molecular weight (MW): Not more than 500 g/mol, (2) Log *p* (octanol–water partition coefficient): Log *p* is a measure of lipophilicity which indicates the ability of a compound to dissolve in fat compared to water, (3) number of hydrogen bond donors: Not more than 5 (total number of nitrogen or oxygen atoms bonded to one or more hydrogen atoms), (4) number of hydrogen bond acceptors: Not more than 10 (total number of nitrogen or oxygen atoms), (5) molar refractivity (MR) provides information on the size and polarizability of the molecule which can affect how the ligand interacts with the protein target in molecular docking. MR values are in the range of 40–130 cm^3^/mol [81]. Based on Tables 4 and 5, the cinnamic acid (**1**) ligand and all its derivatives do not violate from all Lipinski rules. This indicates that all these ligands have the potential to be good oral drugs. In addition, all these ligands can also be categorized as compounds with the potential in strong permeability and solubility.

Toxicity classification, or toxicity class, is a system used to categorize chemicals based on their acute toxicity, typically measured by the LD_50_ (lethal dose for 50% of the test population). There are five categories in LD_50_, namely, Category 1: Extremely toxic (LD_50_ ≤ 5 mg/kg); Category 2: Highly toxic (5 < LD_50_ ≤ 50 mg/kg); Category 3: Toxic (50 < LD_50_ ≤ 300 mg/kg); Category 4: Harmful (300 < LD_50_ ≤ 2000 mg/kg); Category 5: May be harmful (2000 < LD_50_ ≤ 5000 mg/kg); Not Classified (LD_50_ > 5000 mg/kg) [33, 82]. Based on Tables 4 and 5, the predicted LD_50_ value is in Category 5, where all these compounds or ligands are categorized as possibly harmful if swallowed belong to the low oral drug toxicity category.

## 4. Conclusion

Compound **1** as cinnamic acid which was successfully isolated from the leaves of *P. betle* has antioxidant activity that is classified as strong and very strong when tested using two in vitro test methods. In line with the DFT results, this compound has the good antioxidant/antiradical activity in the calculation of Ra/Rd and DAM plots and the deviation in the energy gap from E_HOMO_ and E_LUMO_ is quite small. Similarly, the molecular docking analysis of cinnamic acid (**1**) and its derivatives compounds works on the active side of the amino acid residues of free radical–producing receptor although the binding affinity value is not as strong as the positive control (quercetin). In addition, this study also analyzed oral bioavailability, where cinnamic acid (**1**) and its derivatives can be used as lead compounds in further drug discovery. This study successfully isolated an antioxidant compound from *P. betle* leaves and identified it as cinnamic acid (**1**) through detailed spectra analysis. The findings highlight the potential of *P. betle* as a source of bioactive compounds and underscore the significance of cinnamic acid (**1**) in antioxidant therapy. While further studies involving cellular or animal models may be beneficial, the current study presents a foundational understanding of cinnamic acid's bioactive properties, suitable for preliminary publication.

## Figures and Tables

**Table 1:** Concentration variation of sample test solution.

No.	Concentration (ppm)	Tested solutions (mL)		
		Sample	Methanol	DPPH
1	0	0	2.4	0.6
2	20	0.6	1.8	0.6
3	40	1.2	1.2	0.6
4	60	1.8	0.6	0.6
5	80	2.4	0	0.6

**Table 2:** Antioxidant activity of cinnamic acid (**1)** and methanol extract using DPPH and mSOD method.

Compound	Methods	
	IC_50_ ± SD (μg/mL) DPPH	IC_50_ ± SD (μg/mL) mSOD
Cinnamic acid **(1)**	76.46 ± 2.85	34 ± 1.73
Quercetin (control)	3.88 ± 0.21	5 ± 0.19
Methanol extract	6.87 ± 0.31	9 ± 0.26

*Note:* SD: standard deviation (based on three independent experiments).

**Table 3:** Calculation results in GDP, electron power, index acceptor, and index donor of cinnamic acid.

Compound or atom	Parameter (eV)										
	IP	EA	η	S	Χ	Μ	ω	ω^−^	ω^+^	Ra	Rd
Cinnamic acid (**1**)	7.42	2.64	9.56	5.03	0.05	−5.03	12.65	8.11	3.01	1.47	2.3
Quercetin (control)	9.43	1.75	3.83	0.13	5.59	−5.59	15.64	7.35	1.76	0.84	2.10
Natrium	5.42	0.44						3.50	0.57	0.27	1.00
Florin	15.24	1.95						10.69	2.09	1.00	3.05

Note: η: hardness; *S*: softness; *χ*: electronegativity; *μ*: chemical potential; *ω*: electrophilicity; ω^−^: electron-donating power; ω^+^: electron-donating power; Ra: index acceptor; Rd: index donor.

Abbreviations: IP, ionization potential; EA, electron affinity.

**Table 4:** Inhibition constant (Ki) against xanthine oxidase (XO), NADPH oxidase (NO), cytochrome P450 (CP450), lipoxygenase (LO), predicted lethal dose 50%, and Lipinski's RO5 of cinnamic acid (**1**) and cinnamic group.

Ligand	*Ki* (μM)				LD_50_ (mg/kg)	Lipinski's rule of five					
	XO	NO	CP450	LO		MW	HBD	HBA	LogP	MR	Violation
Cinnamic acid (**1**)	13.38	49.18	109.66	390.11	2500	148.16	1	2	1.78	43.11	0
*o*-Coumaric acid (**2**)	5.01	27.5	57.97	319.28	2850	164.16	2	3	1.49	45.13	0
*m*-Coumaric acid (**3**)	8.87	36.78	60.96	357.69	2980	164.16	2	3	1.49	45.13	0
*p*-Coumaric acid (**4**)	17.75	29.55	53.16	311.37	2850	164.16	2	3	1.49	45.13	0
2,3-Dihydroxycinnamic acid (**5**)	3.05	51.83	31.1	518.35	2980	180.16	3	4	1.2	47.16	0
Caffeic acid (**6**)	8.62	36.64	31.97	480.82	2980	180.16	3	4	1.2	47.16	0
2,4,5-Trihydroxycinnamic acid (**7**)	4.04	21.03	63.54	358.48	2980	196.16	4	5	0.9	49.18	0
3,4,5-Trihydroxycinnamic acid (**8**)	7.02	68.58	51.77	858.48	2980	196.16	4	5	0.9	49.18	0

*Note:* MW: molecular weight (g/mol); HBD: number of hydrogen bond donors; HBA: number of hydrogen bond acceptors; LogP: octanol/water partition coefficient; MR: molecular refractivity (cm^3^/mol).

**Table 5:** Inhibition constant (*Ki*) against xanthine oxidase (XO), NADPH oxidase (NO), cytochrome P450 (CP450), lipoxygenase (LO), predicted lethal dose 50%, and Lipinski's RO5 of ester cinnamic group.

Ligand	*Ki* (μM)				LD_50_ (mg/kg)	Lipinski's rule of five					
	XO	NO	CP450	LO		MW	HBD	HBA	LogP	MR	Violation
Methyl cinnamate (**9**)	23.41	26.83	71.18	50.38	2610	162.19	0	2	1.87	47.43	0
Ethyl cinnamate (**10**)	11.87	18.57	58.33	44.67	4000	176.21	0	2	2.26	52.24	0
Butyl cinnamate (**11**)	4.61	11.46	27.36	29.28	5000	204.27	0	2	3.04	61.85	0
Allyl cinnamate (**12**)	8.12	13.91	40.21	38.33	1520	188.22	0	2	2.43	56.57	0
Phenyl cinnamate (**13**)	0.492	2.23	2.98	9.36	2610	224.26	0	2	3.31	67.55	0
Cinnamyl cinnamate (**14**)	0.448	0.45	0.47	3.48	4200	264.32	0	2	3.96	81.85	0

*Note:* MW: molecular weight (g/mol); HBD: number of hydrogen bond donors; HBA: number of hydrogen bond acceptors; LogP: octanol/water partition coefficient; MR: molecular refractivity (cm^3^/mol).

## Data Availability

The data that support the findings of this study are available from the corresponding author upon reasonable request.

## Supporting Information

All original spectral data supporting the structure elucidation of Compound 1 (UV-Vis, FT-IR, NMR, and MS) are included as Supporting Information. Supporting Figures S1–S8 present the original spectra, and Supporting Tables S1–S3 provide additional data interpretation related to the compound's characterization.
