# Supplementary material for: Density Functional Theory, Molecular Docking Study, and In Vitro Antioxidant Activity of Cinnamic Acid Isolated From Piper betle Leaves
Source: Biochem Res Int. 2025 Jun 17;2025:1691257. doi: 10.1155/bri/1691257 (PMC12187441; doi:10.1155/bri/1691257)
Supplement: Supporting Information — Additional supporting information can be found online in the Supporting Information Section. [file 1691257.f1.pptx]

## Slide 1
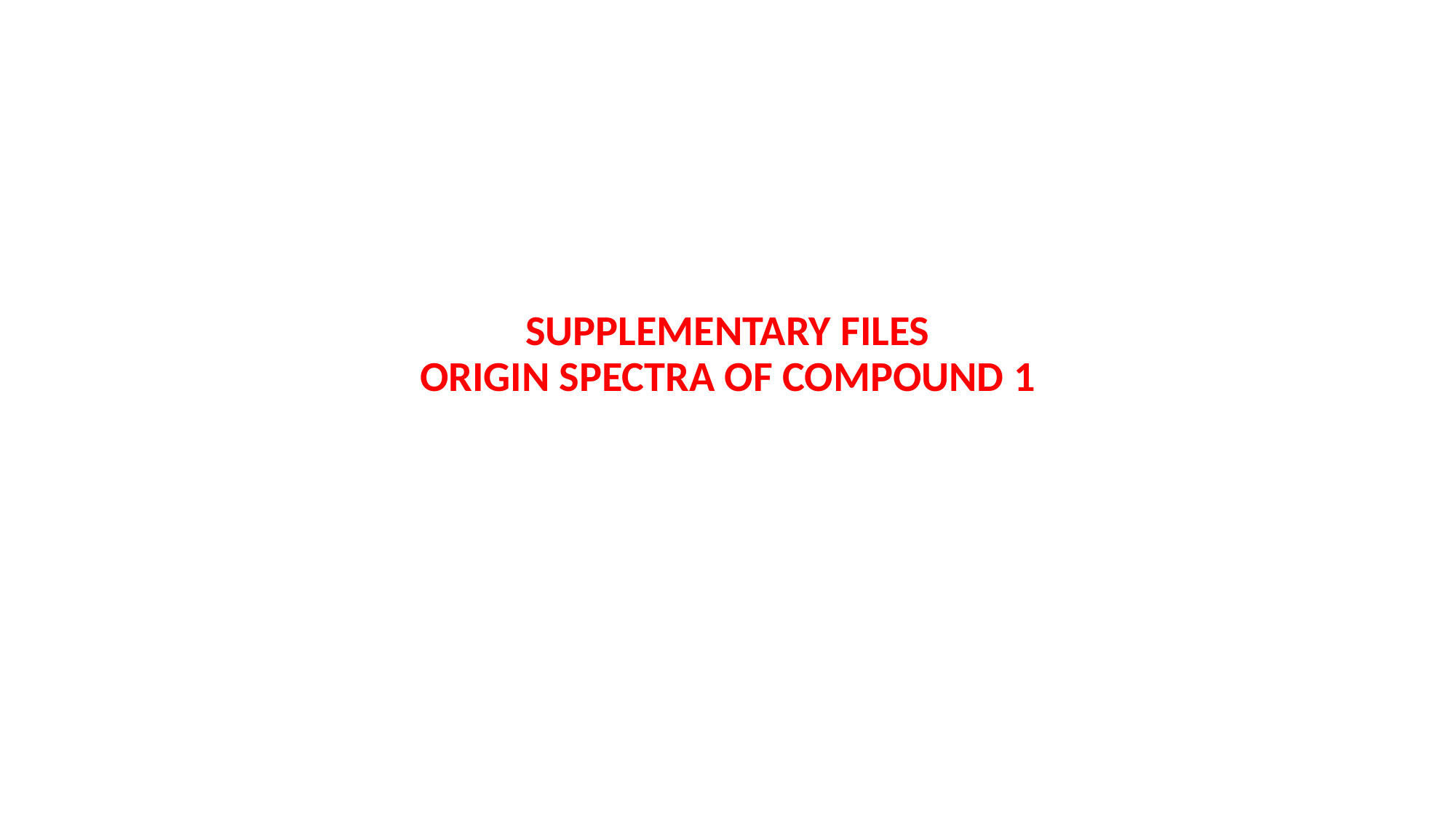

SUPPLEMENTARY FILES
ORIGIN SPECTRA OF COMPOUND 1

## Slide 2
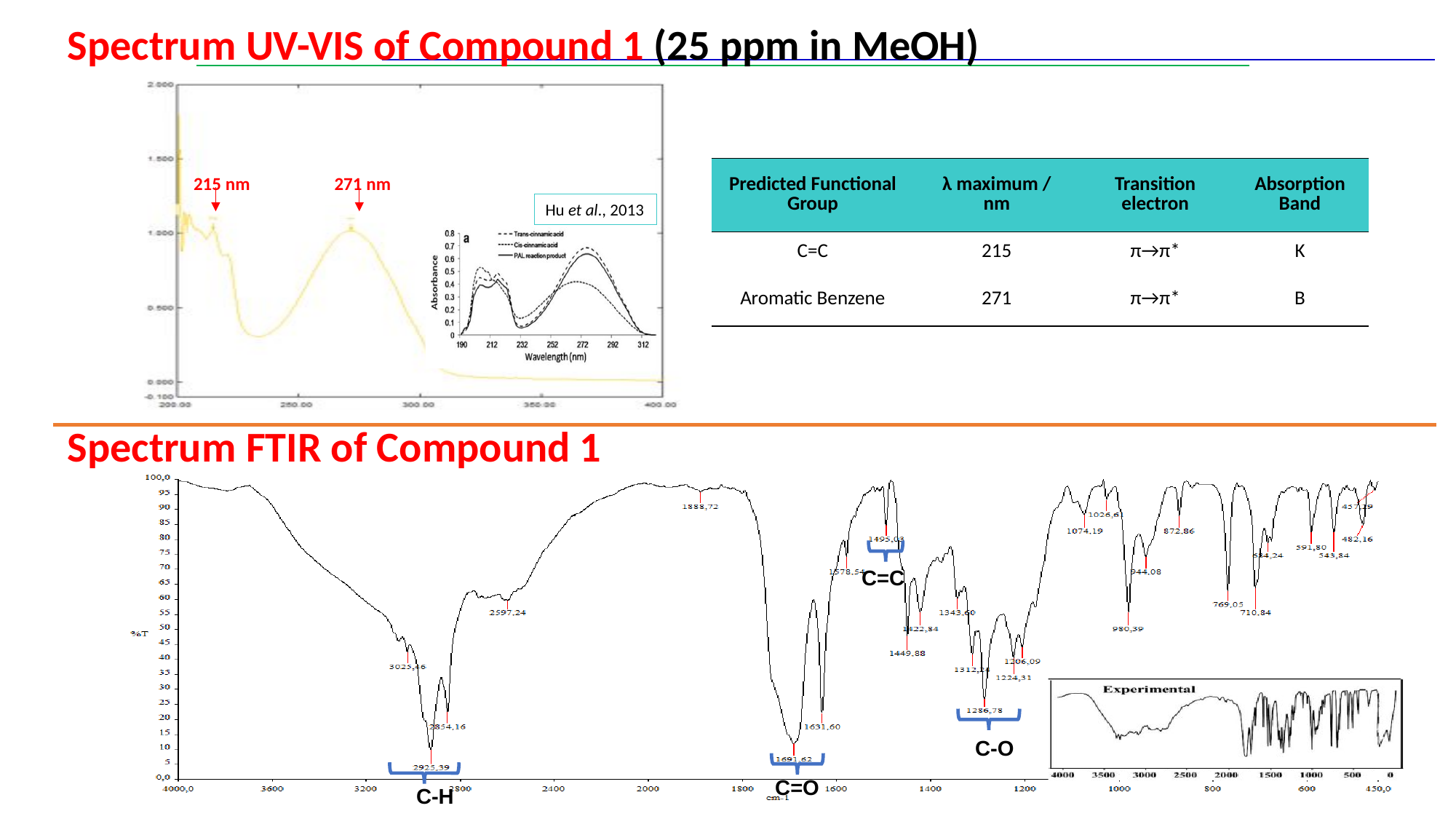

Spectrum UV-VIS of Compound 1 (25 ppm in MeOH)
215 nm
271 nm
| Predicted Functional Group | λ maximum / nm | Transition electron | Absorption Band |
| --- | --- | --- | --- |
| C=C | 215 | π→π\* | K |
| Aromatic Benzene | 271 | π→π\* | B |
Hu et al., 2013
Spectrum FTIR of Compound 1
C=C
C-O
C=O
C-H

## Slide 3
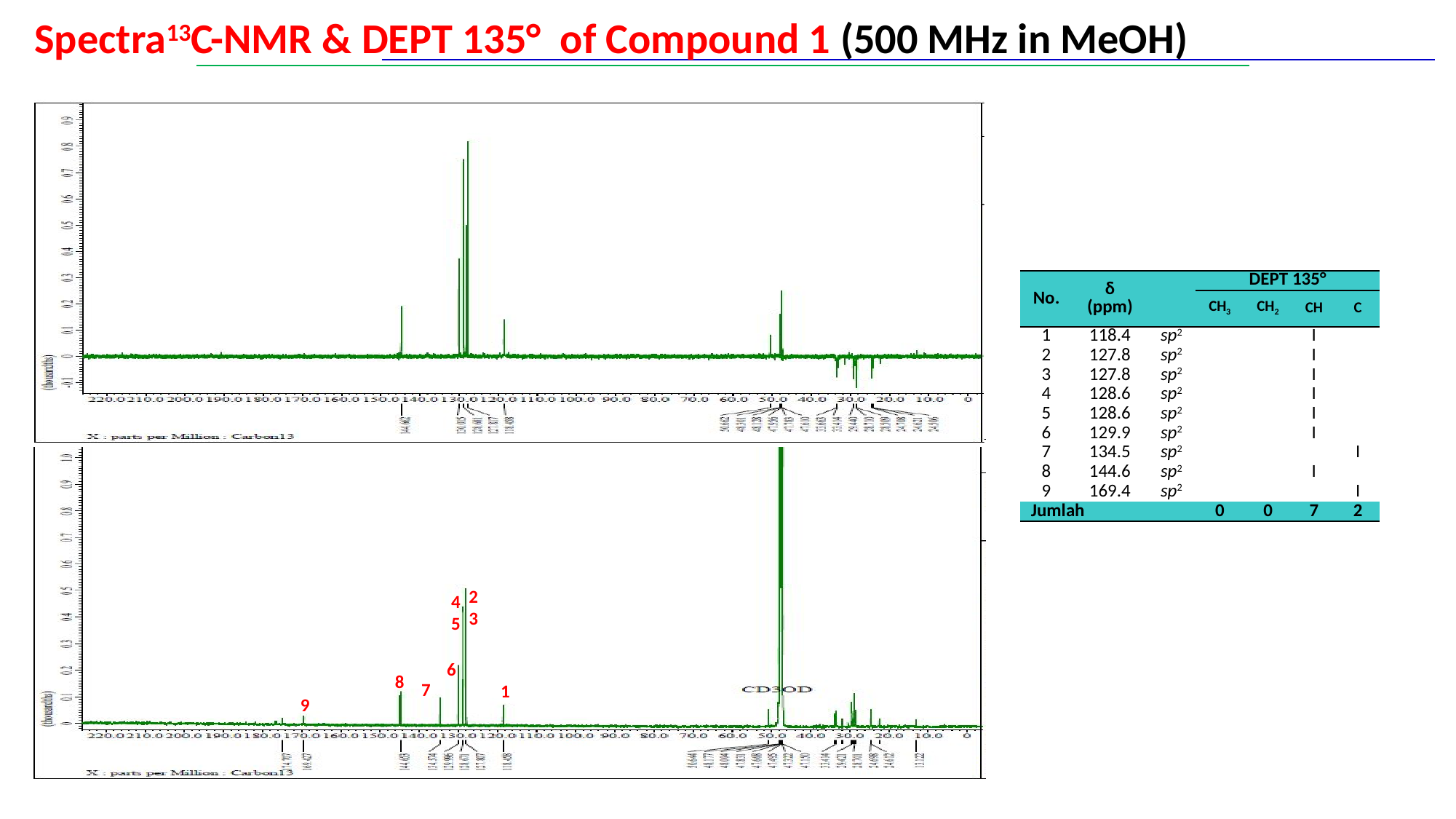

Spectra13C-NMR & DEPT 135° of Compound 1 (500 MHz in MeOH)
2
3
4
5
6
8
7
1
9
| No. | δ (ppm) | | DEPT 135° | | | |
| --- | --- | --- | --- | --- | --- | --- |
| | | | CH3 | CH2 | CH | C |
| 1 | 118.4 | sp2 | | | I | |
| 2 | 127.8 | sp2 | | | I | |
| 3 | 127.8 | sp2 | | | I | |
| 4 | 128.6 | sp2 | | | I | |
| 5 | 128.6 | sp2 | | | I | |
| 6 | 129.9 | sp2 | | | I | |
| 7 | 134.5 | sp2 | | | | I |
| 8 | 144.6 | sp2 | | | I | |
| 9 | 169.4 | sp2 | | | | I |
| Jumlah | | | 0 | 0 | 7 | 2 |

## Slide 4
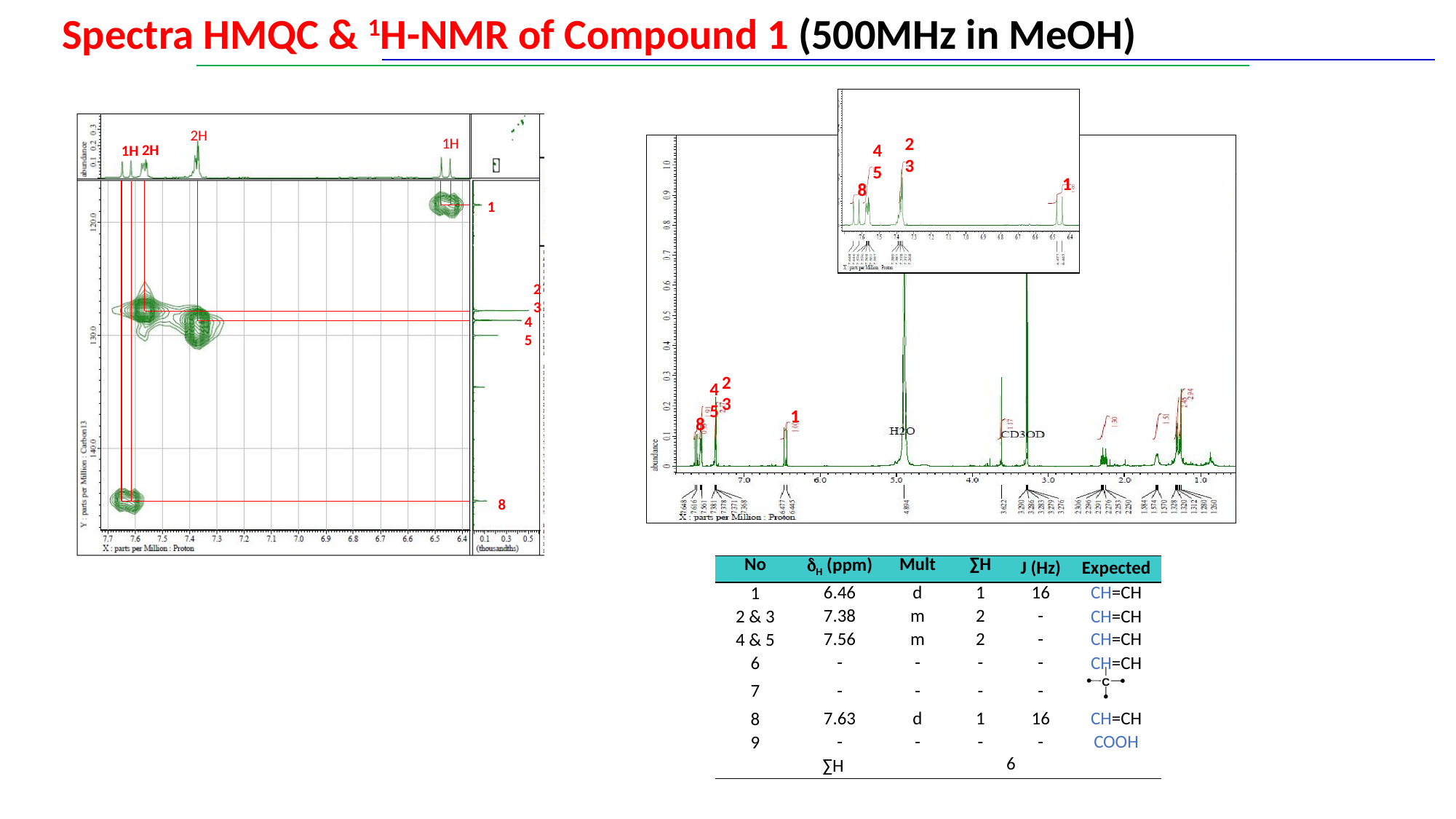

Spectra HMQC & 1H-NMR of Compound 1 (500MHz in MeOH)
2
3
4
5
1
8
2
3
4
5
1
8
1
2
3
4
5
8
2H
1H
2H
1H
| No | H (ppm) | Mult | ∑H | J (Hz) | Expected |
| --- | --- | --- | --- | --- | --- |
| 1 | 6.46 | d | 1 | 16 | CH=CH |
| 2 & 3 | 7.38 | m | 2 | - | CH=CH |
| 4 & 5 | 7.56 | m | 2 | - | CH=CH |
| 6 | - | - | - | - | CH=CH |
| 7 | - | - | - | - | |
| 8 | 7.63 | d | 1 | 16 | CH=CH |
| 9 | - | - | - | - | COOH |
| ∑H | | | 6 | | |

## Slide 5
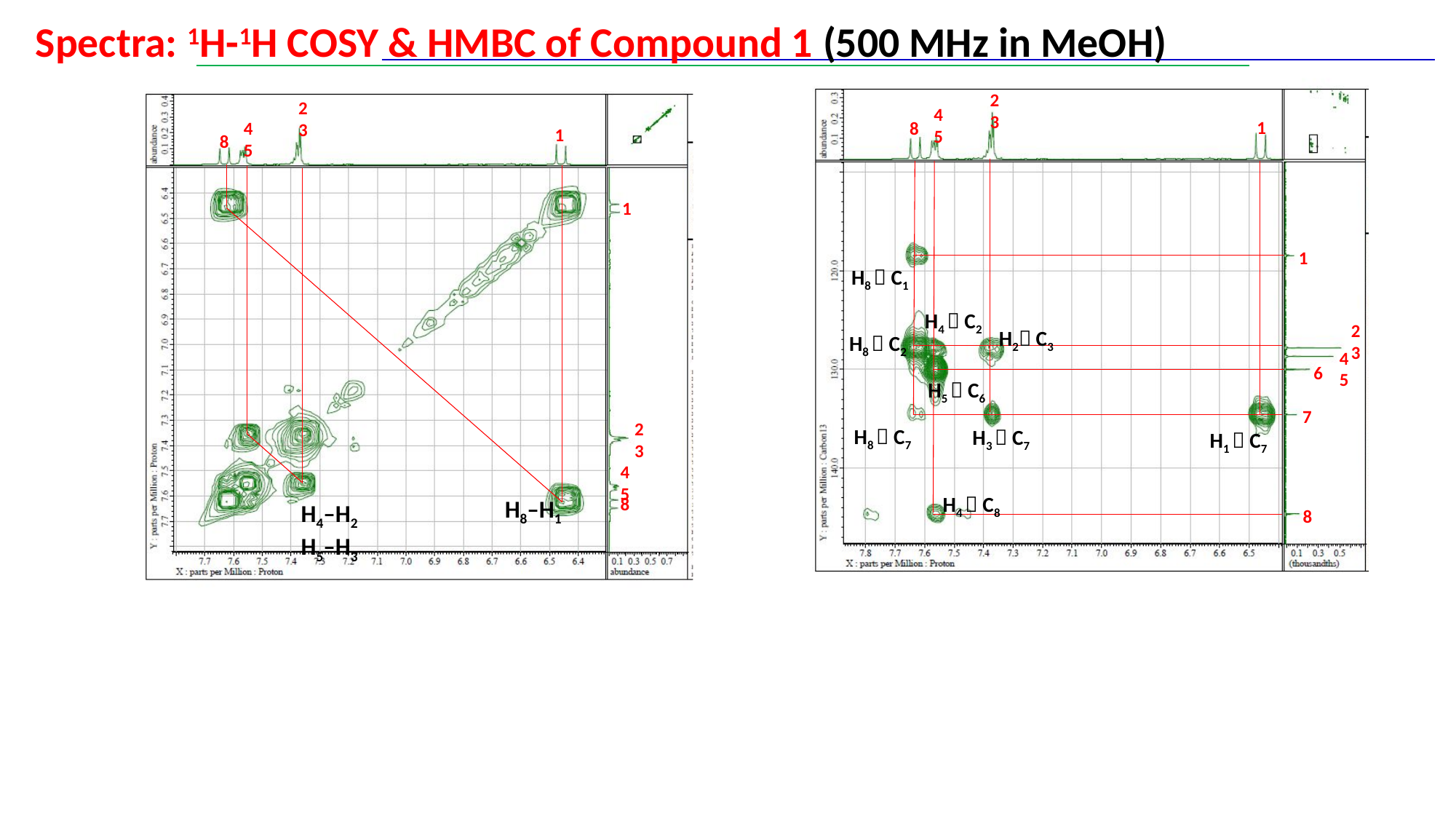

Spectra: 1H-1H COSY & HMBC of Compound 1 (500 MHz in MeOH)
2
3
4
5
1
8
1
H8  C1
H4  C2
2
3
H2 C3
H8  C2
4
5
6
H5  C6
7
H8  C7
H3  C7
H1  C7
H4  C8
8
2
3
4
5
1
8
1
2
3
4
5
8
H8–H1
H4–H2
H5–H3
H1–H8

## Slide 6
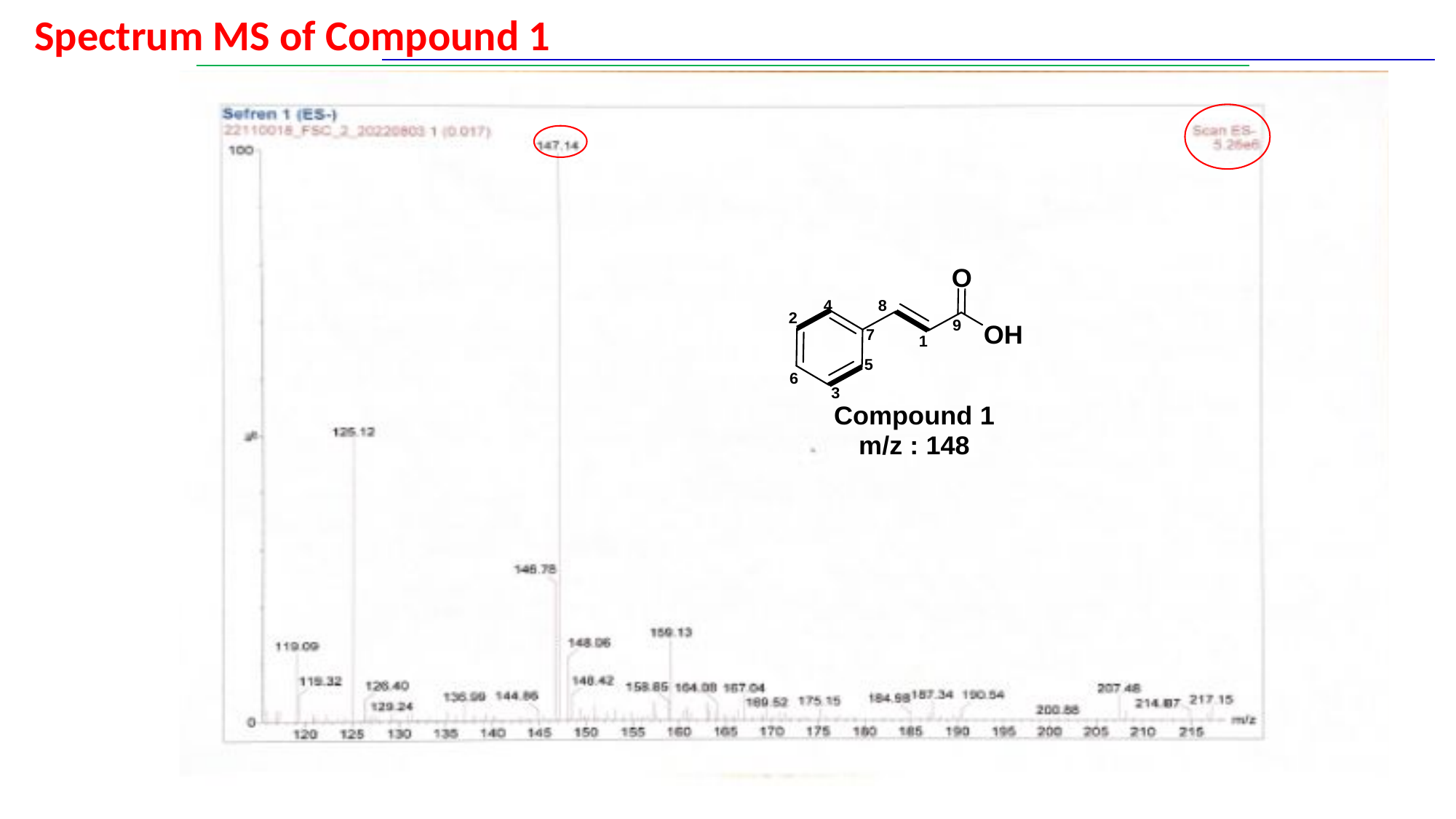

Spectrum MS of Compound 1
